# Exploring the wild almond, *Prunus arabica* (Olivier), as a genetic source for almond breeding

**DOI:** 10.1007/s11295-024-01668-4

**Published:** 2024-09-24

**Authors:** Hillel Brukental, Adi Doron-Faigenboim, Irit Bar-Ya’akov, Rotem Harel-Beja, Taly Trainin, Kamel Hatib, Shlomi Aharon, Tamar Azoulay-Shemer, Doron Holland

**Affiliations:** 1 Fruit Tree Sciences, Volcani Center, Agricultural Research Organization, Newe Ya’ar Research Center, Ramat Yishay, Israel; 2 The Robert H. Smith Institute of Plant Sciences and Genetics in Agriculture, Faculty of Agriculture, Hebrew University of Jerusalem, Rehovot, Israel; 3 Department of Vegetable and Field Crops, Institute of Plant Sciences, Agricultural Research Organization, Volcani Center, Rishon LeZion, Israel

**Keywords:** GWAS, Self-compatibility, Interspecific-population, Kernel size, QTL mapping

## Abstract

During the process of almond (*Prunus dulcis*) domestication, essential traits, which gave plants the plasticity for facing unstable environmental conditions, were lost. In general, the domestication process often narrows the natural genetic diversity. Modern selections (i.e., breeding programs) dramatically accelerated this genetic bottleneck trend to a few successful almond cultivars, which are presently the founders of most commercial cultivars worldwide. The concept of utilizing wild species as a source for important traits and for the enrichment of the gene pool was deeply discussed in previous studies. However, in almonds and other *Prunus* species, deliberate utilization of wild species as a genetic resource for breeding programs is quite rare. To address these significant challenges, we generated an interspecific F1 population between the Israeli almond cultivar Um el Fahem (UEF) and a specimen of a local wild almond species, *Prunus arabica* (*P. arabica*), originating from the Judea desert. This interspecific F1 population possesses high phenotypic variability, and sixteen segregating traits were phenotyped. Among the segregating traits, we were able to genetically associate six agriculturally important traits, such as leaf chlorophyll content (LCC), flower size, and fruit size. The alleles for Self-Compatibility (SC) and kernel bitterness were previously mapped in almond and were reexamined on the background of the distinctive wild genetic material of *P. arabica*. Finally, phenotypic interactions between traits were suggested, such as rootstock perimeter and canopy area that were positively correlated with total yield in the F1 population. This study is a first step towards developing a well-characterized almond interspecies genetic population. The availability of such a genetic tool with detailed phenotypic analysis is crucial to address and explore the profound influence of almond wild species in *Prunus* genetic research and breeding. By using the interspecific population as the infrastructure, we show the advantages and importance of utilizing wild relatives.

**Supplementary information:**

The online version contains supplementary material available at 10.1007/s11295-024-01668-4.

## Introduction

The ability of humankind to domesticate plants is considered the central tier that allowed humanity to thrive (Abbo and Gopher 2017). By domestication, the plant, naturally programmed for survival, was shifted to “serve” production. Nonetheless, this shift for production caused important traits, as adaptation to wide range of climates, to be lost (Hawtin et al. 1996). In the last century, cultivars' improvement evolved into well-organized breeding programs.

Domestication and particularly breeding caused a dramatic decrease in the genetic variance of crops due to many generations of selections (Mariette et al. 2010). However, this genetic variance is the most important factor for the breeder to be able to develop new cultivars with desirable traits. Likewise, this variance is essential for genetic research; the ability to genetically map traits rely first and foremost on genetic variance (e.g., segregated population). Genetic bottleneck is a major problem also in fruit tree breeding. Trees are generally characterized as heterozygous and with high polymorphism. Yet, due to very long generation time (i.e., from seed to seed), most breeding programs were based on two fine cultivars as the starting material, hence rapidly generating genetic bottleneck. In almond, two pronounced reasons enhanced the lack of genetic variance. 1. According to the literature, the mutation that generates sweet kernel and allows almond domestication occurred in a restricted geographic region and was later distributed to other regions (Browicz and Zohary 1996). 2. The main genetic source for self-compatibility, a threshold character in all currently almond cultivars, originated from the ‘Touno’ variety or from one of its descendants. Altogether, the almond cultivated gene pool is very narrow, as recently suggested by a comprehensive study, which showed that most of the almond cultivars grown in the entire world are based on only three sources (Pérez de Los Cobos et al. 2021).

Using wild species as parents for breeding programs is a typical strategy for broadening the gene pool in many annual crops (Hawkes 1958; Tanksley and McCouch 1997; Alseekh et al. 2013). The genetic bottleneck in fruit trees, particularly in the *Prunus* genus, has been discussed previously (Gradziel et al. 2001; Gradziel et al. 2022; Pérez de Los Cobos et al. 2021), and interspecies crosses were suggested for widening almond genetic variance (Gradziel et al. 2001). One example is peach (*Prunus persica*) X almond (*P. dulcis*) introgression lines population (Serra et al. 2016). Furthermore, interspecific crosses are also a common method in rootstock breeding (Vahdati et al. 2021). However, exploiting wild species as parents in cultivar breeding and tree genetic studies is relatively uncommon (Gradziel 2020).

*Prunus arabica* (Olivier), known also as *Amygdalus arabica Olivier* (https://grinczech.vurv.cz/gringlobal/taxon/taxonomydetail.aspx?id=104740, https://www.ipni.org/n/729454-1/, (Shmida et al. 2013)), belongs to the Rosaceae family, *Prunus* genus, subgenus *Amygdalus*, and section *Spartioides* (Browicz and Zohary 1996). It is a wild almond species native to the Asia-temperate zone, including the Zagros Mountains range from southeastern Turkey and northwestern Iran, generally along the Iran- Iraq border. In the Middle East, it can be found in Lebanon (the Anti-Lebanon Mountains), Syria (West Syria: Anti-Lebanon Mountains, the Syrian Desert), Israel (Judean Desert) and Jordan (Northeast and west-central Jordan). *P. arabica* is also found in the Arabian Peninsula and naturally grows in Oman (North Oman: Musandam Peninsula, Al Hajar Mountains), Saudi Arabia (North Saudi Arabia: Harrat Al-Harrah), the United Arab Emirates (North), and Afghanistan (https://grinczech.vurv.cz/gringlobal/taxon/taxonomydetail.aspx?id=104740, (Shmida et al. 2013)). In several countries, including Saudi Arabia, Jordan, Lebanon, Syria and Israel, the taxon is considered a very rare and endangered species (Shmida and Cohen 1989; Sapir et al. 2003; Shmida et al. 2013; El-Sheikh et al. 2019). *P. arabica* is considered to be highly tolerant to drought due to its natural growing conditions and plant morphological, physiological, and biochemical characteristics such as leaves surface reduction and deep rooting system. In addition, exposure of explants to water stress conditions induced by sorbitol and polyethylene glycol indicated that *P. arabica* seedlings survived stress better than other almond species (Sorkheh et al. 2009; Sorkheh et al. 2011). Besides, its natural habitats are diverse and include deserts on one hand, and mountainous regions with harsh cold winters on the other hand, suggesting an ability to well adapt for a high range of climates.

The cultivated almond *Prunus dulcis cv.* Um el Fahem (UEF) is an Israeli leading cultivar for many years. This cultivar was described by D. Barak and S. Adawi in 1959 (Barak and Adawi 1971; Holland et al. 2016), as a random seedling in a local orchard. The UEF is thought to be well adapted to the Israeli environment, with low water and relatively low chilling requirements (Holland et al. 2006). Although this cultivar is self-incompatible (SI), it was a preferred parent in Israeli breeding projects due to its ability to develop flowers on one year old branches, relatively short period for entering the productivity phase, high-quality kernel features and adaptability to the local climate. UEF demonstrates a large sweet kernel with a paper shell (Holland et al. 2006). It is a unique cultivar found in Israel and appears to be genetically distinctive from cultivated almonds around the world (pedigree-based information; Pérez de Los Cobos et al. 2021). As such, it provides a fascinating genetic background for exploring new genetic resources for breeding.

Recently, we published the interspecific UEF x *P. arabica* F1 population (Brukental et al. 2021). There, we demonstrated this population's potential in identifying and mapping the stem photosynthetic capability (SPC) trait. The segregating population is a very effective framework for asking scientific questions and may serve as an infrastructure in almond genetic research. Whole genome sequencing for both parents and the construction of two genetic maps have established a robust infrastructure for this research (Brukental et al. 2021; Trainin et al. 2022).

The main objective of this study is to investigate the use of a wild species *P. arabica* in almond genetic research and breeding. To elucidate the genetic inheritance of a wide range of agricultural traits segregated within the UEF x *P. arabica* F1 population, for understanding the *P. arabica* potential as a parent in breeding. Finally, to genetically associate some of the segregating traits for future marker-assisted breeding or advanced genetic research. The results of the current work emphasize the advantages of using wild species as starting material and its importance for developing new cultivars.

## Materials and methods

### Plant material

The F1 population was generated by crossing between the wild *P, arabica* (male), and the Israeli leading commercial cv. UEF (female) during winter 2017. The F1 population consists of 94 offspring growing on their roots, and one copy of each progeny grafted on the Hansen 538 rootstock in addition to two copies of the parents, grafted on GF-677 rootstock. The trees planted in the almond orchard at Newe-Ya'ar Research Centre in 2019, and were five years old during this investigation and had already produced fruit for two consecutive seasons. Newe-Ya’ar is located in the western Yisraeli Valley at 100 m above sea level, characterized by a Mediterranean, subtropical climate. Orchard trees are grown in clay grumusol (vertisol) soil. The entire population was not pruned during the research period, except for taking down a few shoots from the lower trunk of the trees. The orchard is fertigated according to standard commercial orchard protocols.

### Genotyping, linkage analysis, and genome-wide association studies (GWAS)

The genetic infrastructure established for genetic mapping of segregating traits in the F1 population is described in a previous publication by Brukental et al. (2021). Basically, out of 5,000,000 single nucleotide polymorphism (SNP) markers, we selected 5,000 SNPs spanning the entire almond reference genome (https://www.ncbi.nlm.nih.gov/bioproject/553424) in an average interspace of 40 Kb. Through using this infrastructure together with 94 individual progenies we were able to genetically associate six traits: SK, Sf, LCC, EXG, KIDX and FLA. GWAS was calculated by TASSEL 5.2.59 (Bradbury et al. 2007). The set of SNPs was filtered; markers were discarded when missing data was > 8.6%, and the allele frequency was set for 0.2 < x < 0.8 to prevent overestimation impact of rare alleles. The final SNPs panel contained 3686 markers. A general linear model (GLM) was applied for the phenotypic and genotypic intersect data set to test the association. The phenotypic data load into the model was the arithmetic mean where we have repetitive measurements, and the row phenotypic data when we had only one year of measurements.

For linkage analysis, we used the genetic maps previously published and described by Brukental et al. (2021). Basically, we generate one genetic map for each parent, including 1,560 segregating markers in total. For the Quantitative Trait Locus (QTL) mapping, we used the R/qtl package and implemented the one QTL mode (Broman et al. 2003). For the dichotomous phenotypes (SF and SK), the “binary” model available in the package was added. The threshold for significant results was assessed by 1000 permutation test (α = 0.05).

### Phenotypic measurements

The following phenotypic measurements were conducted on the UEF x *P. arabica* F1 population and parental lines:

#### Blooming date (BD)

Blooming date was assessed for two main time points: At 10% and 100% of blooming. Both, the original and grafted population were measured in two successive years (2021-2022).

#### Flower area (FLA)

While the trees bloomed, fully opened flowers (n = 6-10) were sampled from each genotype. The flowers were scanned with a standard paper scanner (CanoSLIDE 120, Canon, USA). Then the images were analyzed with an image-based analyzer (Tomato Analyzer V4.0, USA). The DPI was held for 300. Results are presented in mm^2^.

#### Leaf chlorophyll content (LCC)

Leaf chlorophyll content was measured during spring on fully expanded leaves for one year. From each offspring, ten leaves from ten different stems were measured during the morning (8:00-11:00) (*n*=10) on the east side of the tree. Each leaf was clamped, and LCC was measured with a chlorophyll meter instrument (MC-100 Chlorophyll Meter, Apogee Instruments, USA).

#### Leaf area (LA)

To calculate the leaf area index, ten leaves were collected from ten different stems (the fifth leaf of the annual growth from each stem) from each individual (*n*=10). Leaves were scanned using a standard paper scanner (CanoSLIDE 120, Canon, USA). Leaf area was then measured and calculated from the scanned images using an image-based analyzer (Tomato Analyzer V4.0, USA) at 300 DPI. This analysis was done for two successive years (2018-2019). The results are presented in mm^2^.

#### Net photosynthesis (A)

Net photosynthesis was measured for the entire F1 population for two different seasons, in summer (A_S) at the end of August, and during February in the following winter (A_W). The measurements are presented as µmol CO_2_ m⁻^2^ s⁻^1^. The detailed protocol is fully described in our previous paper (Brukental et al. 2021).

#### Kernel parameters

Several kernel parameters were measured: kernel size (KIDX), kernel weight (KW), and kernel taste ‘sweet kernel’ (SK). During harvesting time (June), ten fruits from each grafted genotype (n = 10) were harvested and dried at room temperature for almost two months. Ten kernels from each decedent were measured for length, thickness, width, and weight. Kernel size is indicated as index (KIDX), calculated by multiplying seed length by seed width and thickness. Seed weight is presented in grams. For quantifying the kernel bitterness, two independent people tasted five fruits from each genotype and graded them on two scales: 1. Highly bitter (*P. arabica*), bitter, or sweet (UEF). 2. Quantitative scale from 1 (very bitter) to 5 (not bitter). Kernel parameters were monitored for one year.

#### Rootstock perimeter (RS)

To evaluate the effect of each genotype on its rootstock growth (Hansen 538 rootstock), the trunk in each line was marked with a nail 10 cm above ground, and the trunk perimeter was measured. This analysis was conducted during November for two successive years (2021-2022) after the trees ceased to grow in autumn.

#### Self-fertility (Sf) allele genotyping, and self-compatibility (SC) phenotyping

Screening for the *Sf* allele was conducted with previously reported Polymerase Chain Reaction (PCR) genetic markers for the *Sf* allele (Holland et al. 2006, 2016). The corresponding primers are positioned in the second exon of the S allele gene and generate a band of 325 bp for the *Sf* allele (Table 1, Online Resource 1).

**Table 1:** Primers for detecting the *Sf* allele in *P. arabica* and the F1 progeny

Primer name	Primer sequence	Target allele	Product size (bp)
SF1F	5'-TCCAAACTGGAGAGAGCTTG-3'	*Sf*	325
SF1R	5'-GAGACTTCCCCCGATTCTTA-3'		

The Sf marker used in this study distinguishes between the presence or absence of the specific *Sf* allele (Online Resource 1). All the individuals that were found positive for the *Sf* allele (~50%) were phenotypically screened for Self-compatibility (SC). Screening was made by covering a few major branches from each individual with a 15-mesh insect proof net and leaving a few uncovered branches from the same tree as a control. Branches were covered during the flowering period while trees were three years old. A year later, only five trees were covered to validate the results.

#### Remote sensing

RPAS-RGB drone measurements were conducted on the grafted population during summer (end of July) 2021, for one year. Water irrigation for the trees was totally cut off for 21 days. After exposing the trees to water deficit as described above, the F1 tree’s canopy area (CAR), and the ‘excess green index’ (EXG) were determined by a drone using sensors. Flights were conducted 30m above ground level, resulting in a ground sampling resolution of ∼0.6 cm/pixel, using a DJI Phantom 4 Pro UAV (Shenzhen, Guangdong, China). The UAV is equipped with a 20 M Pixel RGB camera with wavelengths at 620–750 nm (R, red), 495–570 nm (G, green), and 450–495 (B, blue). Mission planning was conducted using DJI Ground Station Pro (GSP) software. The flight was designed with 80% image overlap along flight corridors. Then, Pix4DMapperPro desktop software (Pix4D SA, Switzerland; http://pix4d.com) was used to generate orthomosaics by implementing a structure-from-motion (SFM) algorithm. Six ground control points (GCP) geolocated with Real-Time Kinematic (RTK) survey precision were used to georeferenced the orthomosaics. The Pix4D processing options were done according to Pix4D’s “3D Maps” template version 4.1.10. Remote Sens. The ExG values were derived from the georeferenced orthomosaic GeoTIFFs that were generated from the UAV flights using the equation previously used (Aharon et al. 2020): ExG = 2 × G − R − B. Plot-level ExG means from UAV’s were created in ArcGIS® 10.6 (ESRI, Redlands, CA, USA). Shapefiles containing annotated single plot polygons were generated. ExG plot means were generated using the Zonal Statistics as a table function in ArcGIS. Following the ExG evaluations, CAR evaluation of the triticale plots was held by applying a threshold value to the ExG monochromatic orthomosaic, which resulted in a binary image, where white pixels represent vegetation, and the black pixels represent the surrounding soil. Here, the Outsu threshold was used (Otsu et al. 1979). The Outsu algorithm provides a single intensity threshold that classifies pixels into two groups, soil and vegetation. In this case, minimizing the variance of the intra-class intensity values classification and segmentation of the vegetation pixels allowed the evaluation of triticale pixels exclusively for the trees canopy area (CAR) evaluations. This procedure was implemented using an image analysis package in ArcMap.

### Statistics

#### Heritability

The broad sense of heritability (H_2_) was calculated using the ‘Adj R square’ value obtained after performing the ANOVA test using the JMP software. The genotype was the independent variable, and the phenotype was determined as the dependent variable. The variance between the replicated measurement of each genotype was designated as the residual (i.e., environmental effect), and the variance between the genotypes was designated as the genotypic effect.

#### Phenotypic distribution

All phenotypic distributions were tested against several possible models explaining the distribution (‘Fit all’ commend in JMP), the best model (according to the AICc and BIC values) was chosen. Distribution was determined normal or not by the 'Shapiro- Wilk’ test for normality.

#### Phenotypic correlation

For phenotypic correlations the multivariate method was used. Some traits required parametric models for correlation (e.g., ‘pairwise’), and some required the un parametric model (e.g., ‘Spearman’). After implementing both models to find a correlation, the ‘Spearmen’ model was chosen since it was more restricted related to its *r* value. Correlation was concluded as significant when α < 0.05.

## Results

### Phenotypic evaluation of the segregating traits

**Fig. 1:**
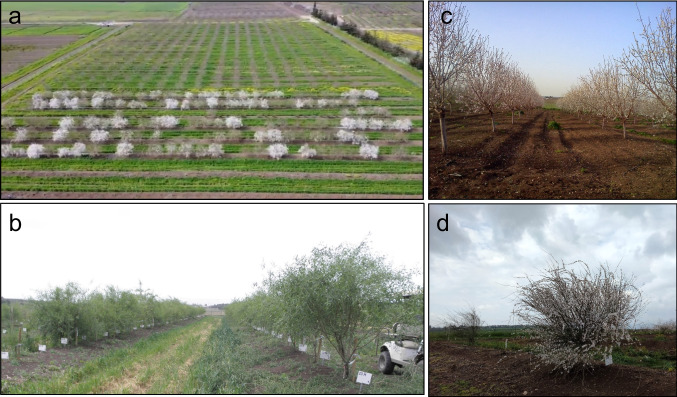
UEF x *P. arabica* F1 population and parents**.** Time of blooming segregated within F1 population (**a**). Tree architecture of the F1 population grafted on the Hansen 538 rootstock (**b**). Typical upright tree architecture of the UEF (**c**), and the bushy *P. arabica* tree architecture (**d**)

The segregating F1 population was generated by crossing *P. arabica* to UEF (Brukental et al. 2021; *n*=94). The population is presented by plants growing on their roots (planted in 2018; Fig. 1a) and a copy grafted on the Hansen 538 rootstock planted a year later (2019, Fig. 1b). The trees are five years old, bearing fruits for two successive seasons. Most progenies flowered in their second year and all of them set fruits already in their third year. BD, LA, RS, TY were monitored for two years, stem photosynthesis (A) was measured during two different seasons (summer, and the follow up winter). All other traits were monitored for one year.

**Fig. 2:**
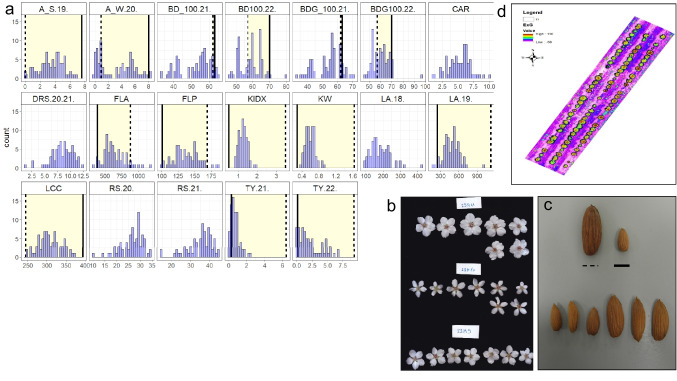
Phenotypic evaluation of the segregating traits in the UEF x *P. arabica* F1 population. Distribution histogram of the phenotypic data for the segregated traits in the F1 population. Y axes are the number of individuals, X axes are the traits’ unit of measuring. Parents’ means are marked by black (*P. arabica*) and dash (UEF) lines in each histogram. The traits include: net summer photosynthesis (A_S), net winter photosynthesis (A_W), blooming date (BD), blooming date in the grafted population (BDG), canopy area (CAR), rootstock perimeter (RS), delta of RS (DRS), leaf area (LA), kernel weight (KW), total yield (TY), leaf chlorophyll content (LCC), flower perimeter (FLP), flower area (FLA), and seed index (KIDX) (**a**). Visual images for representative phenotypic variance are for: flower size of three F1 offspring (**b**), nut size (**c**), black line represents *P. arabica* and dashed line is UEF. Representing map of the F1 population after calculating the Excess Green (ExG) index from the RGB channels (**d**)

The parents of the F1 population differ in their pomological aspects; *P. arabica*, has a small bitter fruit with very hard shell, while UEF has a sweet, relatively large fruit (Fig. 2c) with a ‘paper shell’. Fruit maturation (i.e.,’hull split’) is early in mid-June for *P. arabica* fruits, while the UEF fruit maturation is in mid-August (data not shown). The parents also differ by their tree architecture. UEF has the typical upright growth of a cultivated almond tree (Fig. 1c), while *P. arabica* has a bushy appearance (Fig. 1d). Importantly, since the *P. arabica* used in our study is very rare and only two specimens are found in nature in Israel, it is not clear whether it represents the entire diversity of this species, and its environmental condition in nature. The UEF x *P. arabica* F1 progenies displayed tree architecture that looks as a composition between the parents, with no considerable appearing differences. Fruit maturation date of the F1 did not substantially segregate. All progenies hull split was in the same ± 3 days, at the end of June (data not shown).

Blooming dates were recorded at 10% blooming (BD10) and 100% blooming (BD100) in both growing methods (grafted (BDG) and not (BD)) for two successive years. Both parameters, BD100 and BD10 displayed ‘Normal Two Mixture’ distribution and centered around two main peaks (Fig. 2a, Online resource Table 1) in both years. Heritability estimations for both parameters showed a major genetic component (H_2_= 0.72 and 0.78; Online resource Table 1). Interestingly, the grafted population showed an average delay of about one day in blooming date related to the non-grafted genotypes in both years (BD100 VS BDG100), yet this delay was insignificant (Fig. 2a, Online resource Table 1). Flower size was distributed within the F1 population (Fig 2b). Flower area (FLA) and flower perimeter (FLP) were measured on fully expanded petals of the F1 progenies and their parents. FLP was normally distributed, while FLA was not normal. Transgressive segregation was observed in FLA, FLP and BD parameters (Fig. 2a, Online resource Table 1).

Fruit parameters of shelled nut were evaluated in 2020 for the grafted population. The size and shape of the kernels segregated within the population (Fig 2c). Kernel index (KIDX), represents the kernel size calculated by multiplying height, width and thickness, was normally distributed, while kernel weight (KW) and total nut yield (TY) for both years were not. Relative to TY and KIDX, the average phenotype value among the F1 progenies was lower than the parent’s average, meaning smaller kernels and lower yield (Fig. 2a, Online resource Table 1).

Net photosynthesis of the stems in summer (A_S) was measured during October 2019 and was normally distributed within the F1 population. On the contrary, net photosynthesis of the stems in winter (A_W) showed the ‘Normal Two Mixture’ distribution. Heritability estimation for the SPC trait changed between the seasons and showed significantly higher genetic component in winter (H_2_= 0.91) than in summer (H_2_= 0.65).

Rootstock perimeter (RS) was measured on the grafted F1 population during November for two successive years (2020-2021). Broad sense of heritability for RS showed high value (H_2_= 0.95), and the distribution of the phenotype was not normal in both years. Leaf area (LA) was measured for two successive years (2018-2019). The fifth leaf of the annual growth was measured when fully expanded. Leaf average area was 185 mm^2^ in 2018 but was significantly higher in 2019 (486 mm^2^; Online resource Table 1). Heritability was different between the years, in 2018 it was low (H_2_= 0.47), while in 2019 it was higher (H_2_= 0.62). Leaf chlorophyll content (LCC) was measured during spring on fully expanded leaves and was normally distributed (Fig. 2a). Excessive Green index (ExG) and Canopy area (CAR) were determined by a drone, using RGB imaging. The F1 grafted population was photographed with the RGB channels after exposing the F1 population for three weeks of no irrigation during the heat and dry conditions of July 2021 and presented in this study. CAR and ExG were normally distributed (Fig. 2a, Online resource Table 1).

### Interactions between traits

**Fig. 3:**
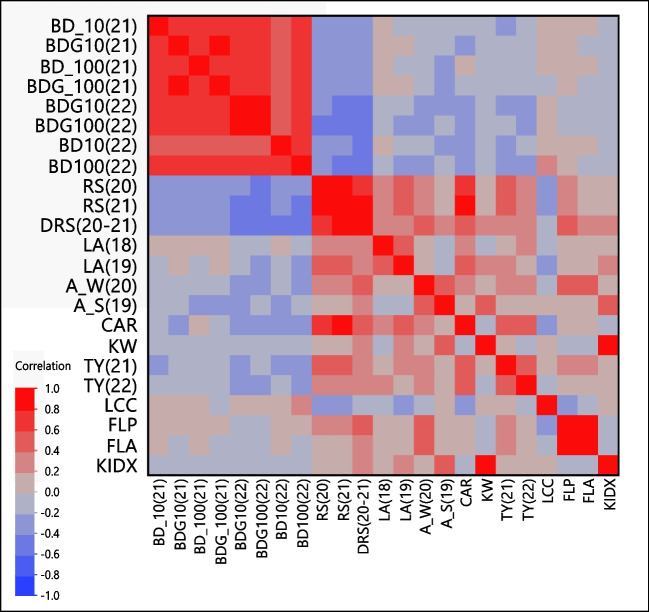
Heat-map of correlations between segregating traits within the UEF x *P. arabica* F1 population. Traits are clustered by their correlation level. The method used for the statistical estimation was Spearman (according to the r value). All the r and p values are detailed in Online resource Table 3. Phenotypes and abbreviation are the same as presented in Fig. 2a: blooming date (BD), blooming date in the grafted population (BDG), rootstock perimeter (RS), delta of RS (DRS), leaf area (LA), net winter photosynthesis (A_W), net summer photosynthesis (A_S), canopy area (CAR), kernel weight (KW), total yield (TY), leaf chlorophyll content (LCC), flower perimeter (FLP), flower area (FLA), and seed index (KIDX). The number in the parentheses refers to the year of measurement

Besides being the infrastructure for genetic mapping, the segregating population may be a framework for understanding physiological interactions between traits. Eighty-five interactions between traits were significant (α < 0.05), among them, 61 were positively correlated (*r* > 0.3) and 24 were negatively correlated (*r* < -0.3). Traits that were evaluated at both years, such as RS20 and RS21 showed a high correlation between the years, (*r* = 0.94, Fig. 3, Online resource Table 2). Blooming date parameters also showed high correlations between the two years of measurements of non-grafted and grafted trees (BD100, *r* = 0.72; BDG100, *r* = 0.69). In addition, the 100% blooming date (BD100) was correlated between the grafted (BDG100) and non-grafted (BD100) progenies (*r* = 0.68) (Fig. 3, Online resource Table 2). However, leaf area (LA) showed a relatively low correlation, but significant, between the two successive years of monitoring (*r* = 0.44, Online resource Table 2, Fig 3). Interestingly, flower size traits (FLP and FLA) showed no significant correlation to KIDX (r= -0.19 α= 0.12 and r= -0.15, α= 0.23, respectively; Online resource Table 2). Stem photosynthesis during winter (A_W) showed a significant positive effect on total yield in 2022 (*r* = 0.39) and a higher effect in 2021 yield (*r* = 0.49). Likewise, A_W demonstrated a positive effect on vegetative vigor parameters such as RS and LA in both years. Surprisingly, A_W showed a high positive correlation with FLA (*r* = 0.58). Nonetheless, stem photosynthesis during summer (A_S) showed no correlation to other traits.

### Genetic association of the segregated traits

**Fig. 4:**
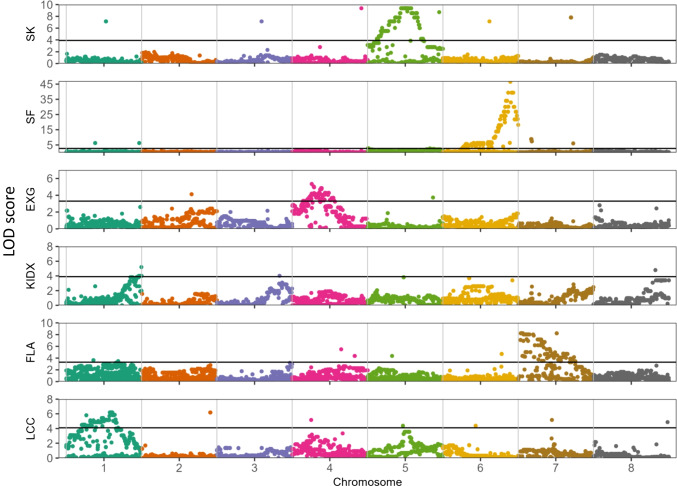
GWAS of six traits within the UEF x *P. arabica* F1 population. The traits are kernel bitterness (SK), self-fertilization (Sf), Excess Green index (EXG), Kernel size (KIDX), Flower area (FLA) and leaf chlorophyll content (LCC). Y axes represent the log of odds (LOD) score, X axes show eight chromosomes, each color representing a chromosome, of the almond reference genome (https://www.ncbi.nlm.nih.gov/bioproject/553424). Horizontal black lines represent the significance threshold for each trait separately (by 1000 permutation tests, α = 0.05)

Elucidating the genetic regions that are regulating traits is essential for developing genetic markers for marker-assisted breeding and for genetic research to understand the mechanism controlling the traits. For the UEF x *P. arabica* F1 population, an efficient genotypic infrastructure of 5,000 targeted SNPs was designed and described earlier (Brukental et al. 2021). For association, we undertook the genome-wide association approach (GWA) with the availability of reference genomes (https://www.ncbi.nlm.nih.gov/bioproject/553424) which allowed us to locate the markers in association with physical genomic position. We determined a signal as a locus in association when the highest marker was part of a "peak" and not an isolated one, and when it was above the normalized significance threshold (determined by 1000 permutation test, α < 0.05). The locus boundaries were defined by ±1 Log of odd (LOD) from the highest marker, or when it was beneath the threshold for significance. Six traits with a potential agronomical impact were significantly associated with genomic regions in the almond genome (Fig. 4; Online resource Table 3). All the traits that showed association with genomic markers were significant with only one major locus. The highest ‘peak’ detected was for the Sf allele (LOD = 47), the trait (i.e., the *Sf* allele presence/ absence) evaluated by screening for the *Sf* allele (Table 1; Online Resource 1) and was positioned at chromosome 6 (25,214,290 to 26,541,014 bp). Accordingly, this locus explained 91% of the phenotypic variance (Fig. 4, Online resource Table 3). The leaf chlorophyll content (LCC) locus at chromosome 1 (24,288,691 to 28,542,875 bp) explained only 29.3% of the phenotypic variance. The locus associated with ExG positioned at chromosome 4 (5,634,098 to 5,878,665), was narrow and spanned ~0.25 Mb, containing 55 genes. Interestingly, the associated region of FLA overlaps with that of the SPC major QTL in chromosome 7, which was previously independently detected by both linkage and association mapping (Brukental et al. 2021). When analyzing the effect of the highest peak SNPs, the *P. arabica* allele demonstrated a positive effect on LCC and SK, while the UEF allele showed a positive effect on FLA, kernel size (KIDX), and ExG. In addition to the GWAS, we conducted a QTL mapping to corroborate and validate our findings. Overall, the six traits show similar signals in the QTL mapping. The LOD score (and permutation threshold) was generally lower in the QTL mapping, which is probably attributed to the shortage of markers in the genetic maps compared to the number of informative markers used in the GWAS. QTL mapping results are plotted in Online Resource 4, and the significant loci are detailed in Online Resource Table 4.

#### Self-compatibility genotyping and functionality in the F1 progeny

The S alleles of *P. arabica* are *Sf* and *S12*, while UEF contains *S7*, *S6* alleles (Bar Yaakov; unpublished data). Moreover, when 856 bp from *P. arabica Sf* allele genomic region (including 522 bp of the exon) was re-sequenced, it blasted against a similar *Sf* allele from the self-compatible cultivar *Prunus dulcis* cv. Lauranne on an unplaced scaffold (https://www.ncbi.nlm.nih.gov/bioproject/553424, AP020489: 31128- 31747; 32149- 32386) with only eleven SNPs (Bar Yaakov; unpublished data). As expected, the marker for the *Sf* allele was segregated in a 1:1 ratio (Online resource 1). Yet, *P. arabica* in the orchard, couldn’t fertilize itself. Self-compatibility was tested for four years using several methods, including hand pollination and covering branches with insect-proof nets. Nonetheless, we tested the ability of self-compatibility in the F1 population and *none of the offspring which contained the Sf allele was self-compatible* (Online resource 1). Therefore, we conclude that this specific *P. arabica Sf* allele is not conferring self-compatibility. Although the SC trait did not segregate, we found it valuable to genetically map the *Sf* allele itself (i.e., the marker presence/ absence), as will be detailed in the discussion.

#### Characterization of the sweet kernel trait in the F1 progeny

**Fig. 5:**
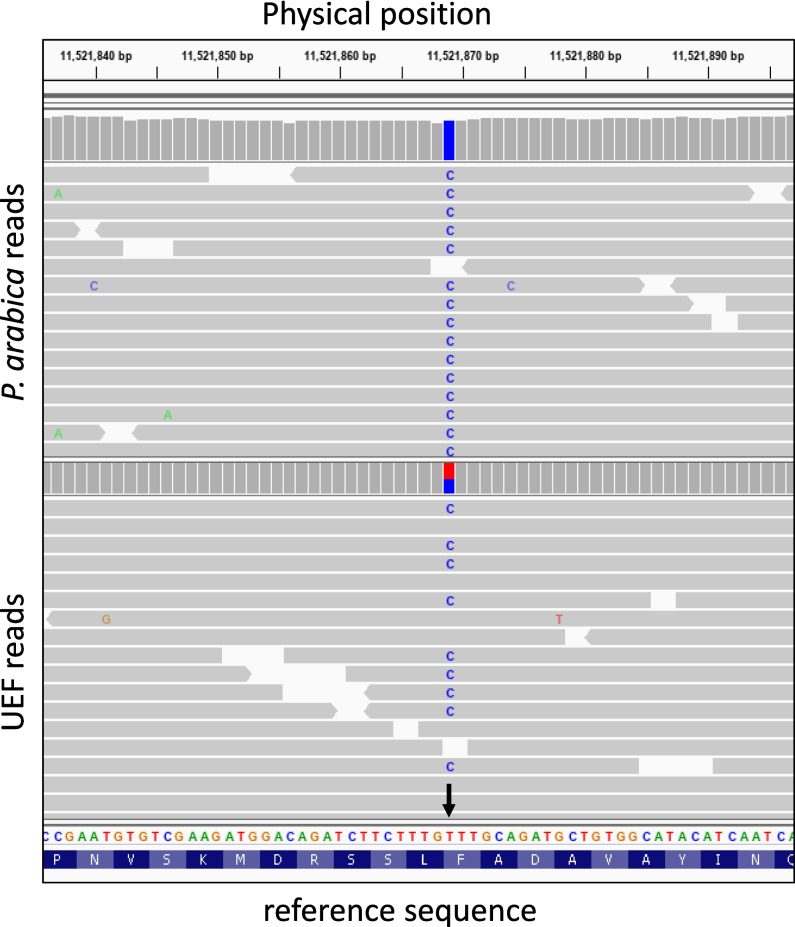
The ‘SK’ SNP mutation in the *P. arabica* and the UEF sequences. Reads of *P. arabica* and UEF mapped against the physical position and against the first exon of BHLH (Prudu_014439) on the ‘Lauranne’ reference genome (https://www.ncbi.nlm.nih.gov/bioproject/553424). The black arrow points to the nucleotide responsible for the ‘SK’ SNP (Sanchez et al. 2019): T/T in sweet ‘Lauranne’, C/T in heterozygote sweet UEF, and C/C in homozygote bitter *P. arabica*. Graphic visualization by IGV v2.5

The distinctive genetic background of *P. arabica* makes it an interesting case for exploring the inheritance nature of the SK trait. *P. arabica* kernels are highly bitter, while UEF kernels are sweet. The bitter or sweet taste of the UEF x *P. arabica* F1 progeny was assessed by organoleptic testing. All F1 progenies had bitter kernels, and none was edible as the UEF. Nevertheless, variability was observed in the level of bitterness among the F1 progeny, which was assessed by discriminating between bitter to high bitter taste and by grading 1 to 5. This segregation was unexpected since the sweet allele was analyzed in other populations as dominant (Dicenta and García 1993). GWAS associated SK trait to the physical position on the almond genome (chromosome 5; 7,554,239 to 11, 832,594 bp) (Fig. 4, Online Resource Table 3), overlapping that published by Sanchez et al. (Sánchez-Pérez et al. 2019). Moreover, a comparison of the genomic sequences of the *BHLH* transcription factor (gene id: Prudu_014439) from UEF and *P. arabica* showed the non-synonymous SNP mutation previously described as distinguishing between bitter and sweet kernel (Dicenta and García 1993; Sánchez-Pérez et al. 2008, 2019; Thodberg et al. 2018). (Fig. 5, Online Resource Table 3). Interestingly, UEF was found to be heterozygous, while *P. arabica* was indeed homozygous for the bitter allele.

## Discussion

### Population general overview

In self-incompatible species such as almond, an F1 population could serve as a mapping population due to the high polymorphism and heterozygosity (Olukolu et al. 2009; Gabay et al. 2018). Moreover, crossing between wild-type and domesticated almonds provides a deeper insight into the genetic nature of traits, including information on their dominant or recessive phenotypes, and whether they are controlled by a single/major gene or by multiple genes (Brukental et al. 2021). Although the F1 population is a cross between two different almond species, the use of *P. arabica* as a male and UEF as the female successfully generated a high percentage of fruit set and a high germination rate (data not shown). Moreover, most of the progenies survived, and all the progenies of the F1 were fertile. The ability to cross between different almond species in the *Prunus* genus is well documented (Gradziel et al. 2001; Gradziel et al. 2022) and the UEF x *P. arabica* F1 population is an additional new example for this capability. Yet, a detailed follow up of the inheritance of traits such as fruit quality and yield in such cross-species populations and in advanced generations of these population is essential for both, breeding of improved cultivars and for understanding the biochemical nature of the traits.

Interestingly, none of the reciprocal crosses succeeded, and it was impossible to obtain a successful pollination when *P. arabica* was used as the female. This unidirectional type of crossing displayed in this combination of genetic cross may suggest a sex dependent lethality of the developmental pathway leading to fruit development in particular species of almond. Such a mechanism could also contribute to the reduction of genetic variability.

Besides the SPC trait that was described recently (Brukental et al. 2021; Trainin et al. 2022), additional traits were segregated within the UEF x *P. arabica* F1 population. Phenological traits, as BD, FLA and FLP (Fig. 1a, Fig. 2a,c), leaf characteristics, as LA, LCC, ExG, and CAR. Kernel characteristics, as KW, KIDX, and SK, and finally, the highly valued commercial traits as yield, and rootstock size (TY and RS respectively (Fig. 2a, Online resource Table 1). This data, obtained only from a single cross population, already points to those traits for which F1 population could be useful for breeding purposes, for genetic analysis and for assessing the difficulties anticipated by using almond biological material from the wild. Usage of exotic genetic background as *P. arabica* could be an important resource for diseases or pests’ resistance (Decroocq et al. 2016). Preliminary data of field observations suggest prominent difference in sensitivity of wild *P. arabica* and its F1 progenies to insects and disease. Both higher resistance and higher sensitivity to biological threats are of importance. Future research of the UEF x *P. arabica* F1 population should definitely take this into consideration, and evaluate this population as a useful genetic resource.

GWAS is presented and discussed as the main approach in this paper, since the segregating population is F1 generation, therefor two different genetic maps, one for each parent, should be analyzed. With the markers that were used for this study, both maps together contain fewer markers as compared to the number of informative markers in the GWAS, as was shown in Brukental et al. (2021). Nowadays, that genomic sequences are quite available, GWAS is a very informative infrastructure. Brukental et al. (2021) demonstrated that within the F1 population there is significantly good collinearity between the genetic order and the reference genome (https://www.ncbi.nlm.nih.gov/bioproject/553424) physical order. However, we performed QTL mapping against the two genetic maps. In general, GWAS and QTL mapping showed similar patterns, which is another evidence of the ‘macro’ collinearity between the reference genome to our genetic maps. For some traits, such as KIDX and LCC, additional minor effect QTLs were detected, probably due to low permutation threshold, which in some cases was beneath LOD of 2 (Online resource 4). Yet, those QTLs explain a relatively low portion of the phenotypic variance (data not shown). Although it is not the main goal of this paper, the two genetic maps allow us to understand better which parent is the donor for the trait variability. Most of our QTLs originated when we mapped the phenotypic data against the UEF genetic map (Online Resource 4). The *P. arabica* low degree of heterozygosity related to UEF (~35 % and 70% respectively; Brukental et al. 2021) may explain that, and maybe advanced populations could segregate new important loci. Interpreting and comparing phenotypic variance explained, LOD, and other QTL mapping results is limited due to the different sets of markers used for each genetic map and for GWAS. One of the traits that intrigued us was the phenotype of tree structure because of the high difference between *P. arabica* and UEF. (Fig. 1c, d). *P. arabica* has a general appearance of a shrub with several major trunks, while UEF has a classical structure of almond with a major trunk and upright growth of green stems. Interestingly, this trait did not segregate within the F1 population, and all the F1 progenies have an overall similar tree structure with characteristic stem ends that tend to bend towards the ground leading to a weeping willow-like appearance (Fig. 1a-d). This trait is requiring advanced populations for genetic studies. Data from other crosses of *P. arabica* with cultivated species do not show the “weeping willow”-like phenotype suggesting that this trait could originate specifically from the combined genetic background of *P. arabica* and UEF. Another trait that did not segregate in the F1 population is the hardiness of the shell. This trait is of high importance as it potentially affects sensitivity of the fruit to insects that attack the kernel and the easiness of mechanical de-shelling of the fruit (Gradziel 2009). All the progenies of the F1 population bear fruit with hard shell similar to that of *P. arabica*, which suggests dominance of this trait at least on this genetic background. This result is in accordance with previous almond mapping populations that showed hard shell dominancy (Sánchez-Pérez et al. 2007a). To further study the genetics of those traits, additional generations should be generated.

### Phenotypic evaluation

Analyzing the segregation of TY, LA, RS, BD, BDG, A_W traits within the F1 population revealed that they demonstrated a significant non-normal distribution, all other traits that were measured showed normal distribution. The SK and Sf traits were measured qualitatively, hence, their distribution was non-normal. Previous studies suggested that the blooming date (BD) trait is a quantitative trait with high heritability, although some populations demonstrate the bimodal distribution (Sánchez-Pérez et al. 2007b). In the F1 population of this study, BD also showed constitutively the bimodal and not a classic normal distribution (Fig. 2a), and relatively high correlation between years (Online resource Table 3). This is in accordance with previous work that study BD in three interspecific population in the *prunus* genus, which demonstrate high correlation between years (Dirlewanger et al. 2012). Furthermore, QTLs for BD were detected constitutive between years. However, within the F1 population association study was not significant, even not to the known genomic loci found in previous studies such as the *DAM* genes in LG 1 or the *LB* in LG 4 (Ballester et al. 2001; Silva et al. 2005; Aranzana et al. 2019). The broad sense heritability we obtained (H_2_= 0.72-0.78) was quite similar to previous reports (H_2_= 0.8; Kester et al. 1977). BD is directly connected to the tree carbohydrates status in dormancy (Sperling et al. 2019). It is possible that the ability of *P. arabica* to assimilate CO_2_ through its stems during winter reveals a new unknown regulation of BD. Noted here is the fact that the blooming date of the parents of the F1 population, *P. arabica* and UEF, occur at about the same time (observation of five years, data not shown), while most of the mapping of BD was reported for populations that had parents with distinctive BD phenotype. LA showed a significant positive correlation between years (Online resource Table 2), but relatively low, suggesting a major environmental effect for this trait. Moreover, there were significant differences in the LA average between years (Online resource Table 2), indicating that tree age should be taken into account in almonds while screening for LA. Interestingly, TY shows a high positive correlation between years (r = 0.59; Online resource Table 2); this consistent phenotype may suggest the reliability of early screening for yield in almond breeding populations.

Through using the F1 infrastructure of 94 individual progenies we were able to genetically associate six traits: FLA, LCC, ExG, KIDX, SK and the *Sf* allele. The ability to genetic associate already in the F1 generation these important traits sets a promising approach for further fine genetic mapping and for marker assisted breeding.

#### Flower area (FLA)

A significant difference in FLA was observed between *P. arabica* and UEF. This phenotype segregated in the F1 population and genetically associated with one major locus located in chromosome 7, explaining 43% of the phenotypic variance (Fig. 2a,b; Fig.4; Online resource Table 3). From a general observation of Newe-Ya'ar almond germplasm (Holland et al. 2006), it seems that there might be a phenotypic correlation between flower size and kernel size in almond. Likewise, UEF, which has a significant bigger FLA than *P. arabica*, bears bigger fruits (Online resource Table 1). Nonetheless, the data from the F1 phenotyping indicates no phenotypic correlation between FLA and KIDX (*r* = -0.15, *p* value > 0.05; Fig. 3, Online resource Table 2). Moreover, GWAS analysis of these two traits-mapped them to different chromosomes (chromosome 1 and chromosome 7 respectively), meaning they are separately and independently inherited (Fig. 3, Fig. 4). Our results indicate that those two traits are not pleiotropic, FLA is not a good physiological marker for fruit size. The genetic background of our F1 population enabled the separation of the genetic regulation of the flower size and fruit size traits.

The locus associated with FLA overlaps with the QTL for SPC, which was demonstrated in our previous study (Brukental et al. 2021). Hence, those two traits are also phenotypically correlated (*r* = 0.58, α < 1.019E^-07^). To our knowledge, no published data points to a possible physiological correlation between photosynthesis or sugar content and flower size. It is possible that on the UEF genetic background, the extra carbon gain in those F1 progenies, harboring the SPC, is expressed in bigger flowers. Given the above, we cannot conclude yet whether the linkage between the traits is only genetic or also phenotypic. Previous study in ornamental *Prunus mume* genetically mapped flower traits as color, shape, and pistil number to a major locus on chromosome 1, yet to the best of our knowledge none of them dealt with FLA as a character (Li et al. 2022).

#### Leaf chlorophyll content (LCC)

Crop growth and development depend primarily on chlorophyll, the most critical photosynthetic pigment that plants use to absorb the energy from light and convert it to carbohydrates. Leaves play as the primary photosynthetic tissue in most plants, and a positive correlation between leaf chlorophyll content (LCC) and its photosynthetic rate has been established (Mae 1997; Guo et al. 2008; Nahakpam 2017; Zhao et al. 2019). Leaf chlorophyll content was found to be tightly related to yield and economic gain (Mauromicale et al. 2006; Quarrie et al. 2006; Gao et al. 2013; Faralli and Lawson 2020). For these reasons, the LCC trait has attracted a lot of interest, and was the center in basic research and breeding programs (Ye et al. 2017; Jian et al. 2020; Lu et al. 2021). In an almond-peach interspecific population, a QTL for LCC was found in LG 6 (Serra et al. 2016). In the current study, analysis of the LCC was found to be significantly higher in *P. arabica* compared to UEF (393.7±11.9 and 243.9±10.8 µmol m^-2^, respectively).

LCC segregated and demonstrated a normal continuous distribution within the F1 progeny. LCC broad sense heritability was moderate (H_2_= 0.43) relative to other traits that were measured (Fig. 2a, Online resource Table 1). These results may suggest that this trait in almond is regulated by multiple genes and is significantly environmentally affected. However, only one locus was detected as significantly associated (Fig. 4). Our results emphasize the advantage of using wild species as donors of important traits such as high chlorophyll content. The locus we detected could potentially be used in future marker-assisted breeding programs. Further research on other genetic backgrounds is needed to cross-validate the locus we detect, test its potential for agronomic contribution in future breeding programs, and identify the gene/s involved in LCC regulation.

#### Excess green index (ExG)

Nowadays, when sequencing techniques are remarkably advanced, with significantly lower cost, the bottleneck in genetics is the ability for high throughput phenotyping allowing rapid screening of high number of plants. In the last years remote sensing technology as LIDAR, thermal imagining, RGB cameras and other sensors became common as a method for collecting big data very efficiently with sensitive resolution. Placing those sensors in a drone or satellite makes the ability for large-scale phonemics even more efficient. Yet, the major step for implicating those methods is calibrating the raw sensor data (pixels, indexes etc.) and translate it to physiological parameters. Here we utilized a simple RGB camera placed on a drone for detecting the response of the individual progenies of the F1 population after exposure to three weeks of drought (i.e., no irrigation during July). The results were transformed to ExG index. The ExG is based on an RGB picture, and related basically for the G signals (i.e., the reflection of the green color in the spectrum), therefore it is considered as an indexed indicator for plant vegetative development and vigor or simply as another parameter of the plant chlorophyll (Aharon et al. 2020). This phenotype revealed a trait, which segregated in the F1 population and was associated to a very convincing QTL in chromosome 4 (Fig. 4). Undermining the physical location of the QTL and the genes within the QTL boundaries could reveal specific candidate genes that could potentially relate to response for drought. Nonetheless, more measurements and appropriate controls will be required to understand the physiological meaning of those results. It is, however, demonstrated here that using drone measurements for remote sensing is efficient in obtaining large-scale phenotypic data for genetic mapping in almond and trees in general. Moreover, we could use the data for screening each of the progeny to drought stress and also check the relevance of the newly found QTL in other populations exposed to drought.

#### Kernel size

Fruit size is an important agronomical trait, both to increase yield, and for higher fruit quality. Wild almonds generally bear small fruits. Hence, they are useful for generating segregating populations to obtain genetic markers for breeding and for understanding the genetic regulation and mechanism behind this important trait. The F1 population mean of the kernel size phenotype (KIDX) was lower and significantly different from the parents' mean (1.24 and 1.9, α< 0.0001), and no transgressive segregation was observed. The segregation showed normal distribution of the KIDX, and its heritability was 0.64 (H_2_) (Fig. 2a, Online resource Table 1). GWAS analysis located the associated locus for KIDX at the end of chromosome 1 (Fig. 4). Previous study detected other QTLs for kernel size parameters, kernel length was mapped on chromosome 7 and kernel weight on chromosome 4 (Pérez de Los Cobos 2022). Another study detected markers in association with kernel length in LG 5 and LG 6 (Font i Forcada et al. 2015). Therefore, we suggest here a novel QTL for kernel size. Elucidating the heritability for fruit size, and more importantly, generating genetic markers based on this GWAS will facilitate the efficient removal of the unfavorable low fruit quality alleles from the wild in future breeding programs.

#### Sweet kernel (SK) and self-compatibility (SC)

Almond SK and SC are two highly studied and important traits (Sánchez-Pérez et al. 2007a). For those two traits the genetic mapping, inheritance and mechanism are well established (Company et al. 2015; Sánchez-Pérez et al. 2019). Both traits are useful for inspecting the F1 genetic infrastructure. In addition, both traits, particularly the research of *Sf*, rely on limited genetic resources (Pavan et al. 2021).

Sweet kernel was one of the crucial characteristics for almond domestication to make it edible for humans. Early genetic studies on several different segregating populations demonstrated a monogenic dominant inheritance of the trait (Heppner 1926; Dicenta and García 1993). Later, the trait was genetically mapped to LG 5 (Sánchez-Pérez et al. 2007a). Metabolic studies revealed that in sweet almond varieties, there is a significant decrease in the expression of two enzymes. These enzymes are responsible for the synthesis of prunasin, which is the precursor of amygdalin (Sánchez-Pérez et al. 2008, 2012; Thodberg et al. 2018). A recent study confirmed the genetic mapping, and located the SK locus on chromosome 5 in the physical map on the ‘Lauranne’ reference genome (Sánchez-Pérez et al. 2019). In this study the authors also demonstrated that a single nucleotide mutation on a *BHLH* transcription factor (gene id: Prudu_014439) is responsible for the reduction in the expression of the two enzymes. Moreover, the authors showed that this SNP is conserved in many different almond cultivars.

*P. arabica* has a very bitter kernel, while UEF kernel is sweet . Remarkably, the sequences of *P. arabica* and of UEF show the same SNP, that was reported by Sanchez-Perez et al. (2019). The allelic composition for the bitter *P. arabica* is C/C and C/T for the sweet UEF (Fig. 5). Yet, although the sweet phenotype was suggested to be dominant in other populations (Heppner 1926; Dicenta and García 1993; Sánchez-Pérez et al. 2007a), none of the offspring in the UEF x *P. arabica* F1 population was sweet as the UEF. Actually, all the progenies of the F1 population were not edible. However, there was segregation between highly bitter (as *P. arabica)* and bitter; far from the UEF sweetness. This segregation genetically mapped to the same published SK locus (Fig. 4, Online Resource Table 3, Online Resource Table 4). It is possible that *P. arabica* distinctive genetic background reveals additional genetic components for the kernel bitterness which were not previously reported. Such might be, for example, some redundancy of the path for amygdalin synthesis that could overcome the mutation in the *BHLH* to produce dominant inheritance of the bitter kernel phenotype. Maybe other components, such as some bitter polyphenols, are involved. If this is the case, advanced populations that use backcrosses might reveal this new bitterness element.

The SC trait was genetically mapped to LG 6, the *Sf* allele responsible for SC was detected, and the gene sequence was established (Ushijima et al. 2003; Fernández i Martí et al. 2010; Fernández i Martí et al. 2014; Gómez et al. 2015; Kodad et al. 2015). *P. arabica* contains the *Sf* (Online Resource 1) and *S12* alleles, and the UEF contains the *S7* and *S6* alleles (Bar Yaakov; unpublished data). Remarkably, although *P. arabica* contains the *Sf* allele, it is not self-compatible. This phenomenon was shown also by others, who found some rare varieties with a phenotype of self-incompatibility despite the presence of the *Sf* allele. The inactive *Sf* allele was designated as *Sfi* (Fernández i Martí et al. 2014). Nonetheless, to this day, the mechanism discriminating between *Sf* and *Sfi* is not clear. It was suggested that a unique methylation that occurs on the *Sf* allele, but not on the *Sfi,* is the cause for the silencing of the *Sf* allele expression (Fernández i Martí et al. 2014). However, the research of the *Sfi* was limited to a specific section of almonds (*Eumigdalus* Spach.), while the *P. arabica* presents a new origin for the *Sf* allele, which is from the different *Spartioides* Spach. almond section (Gradziel et al. 2001). This new *Sf* origin could be an important test case for the *Sf*-dependent methylation hypothesis. In addition, the presence of the *Sf* allele in the wild almond *P. arabica*, and the fact that it is inactive, could indicate that the *Sf* allele was already in a common ancestral almond genome with a ‘normal’ phenotype like that of other *S* alleles (i.e., self-incompatible). Probably, an additional mutation that occurred on the *Sf* allele in *Prunus webii* or its descent, the ‘Touno’ cultivar, resulted in self-compatibility. This mutation on the *Sf* allele is currently the main source for self-compatible commercial almonds. *In fact, in our population, we can only map the Sf allele and not the SC trait itself*; however, we found it valuable for two main reasons: 1. Although the *Sf* allele is well studied and was previously genetically mapped, it was not located on the physical map of the ‘Lauranne’ reference genome (https://www.ncbi.nlm.nih.gov/bioproject/553424). Nonetheless, our F1 genetic association located the *Sf* allele on chromosome six. 2. These results are in accordance with previous genetic mapping (Ballester et al. 1998) and confirm the validity of our genetic infrastructure. The fact that the *Sf* allele did not confer SC makes the current *P. arabica* irrelevant as a source for new SC cultivars. Yet, *P. arabica* could be of importance in phylogenetic studies since it is apparent that the *Sf* allele is frequently found in several different species of wild almond in the Newe-Ya’ar almond collection.

### Physiological interaction

Besides genetic mapping, segregating populations are also a substantial framework for understanding the physiological relationship between traits (Sabag et al. 2021). The phenotypes of the progeny give us the ability to examine what are the interrelations between different traits. Are they genotypically or phenotypically dependent on each other, or do they obey simple mendelian genetic behavior. Rootstock perimeter (RS) is a simple and a common indication for tree vigor, which demonstrated very high correlation with tree canopy area (CAR) as determined by remote sensing by drone (*r* = 0.76 α<0.000001). Leaf area (LA) was positively correlated with RS (*r* = 0.36, α<0.01). Likewise, RS and CAR showed a significant positive correlation with total yield (TY) (*r* = 0.49, α<0.0002). These results emphasize the advantages and capabilities of remote sensing for large scale measurements for tree vigor (Zarate-Valdez et al. 2015). Furthermore, it might be possible that rootstock perimeter can be an early physiological marker for yield in future breeding program. Since rootstock perimeter is a trait that could be determined by remote sensing, these data are quite useful for effective breeding programs. Interestingly, blooming date showed no correlation with total yield (TY; *r*<0.24, α>0.05) or with fruit size (KIDX; r<0.16, α>0.05). Here we detailed just a few interesting correlations for emphasizing the importance of the used infrastructure (i.e., the F1 population) for future physiological research, and also as a pre breeding research to elucidate desirable or undesirable interactions. Nonetheless, it is important to note that the results are correct for the current population and this specific genetic background, and additional research is essential to determine how general the results are.

### Candidate genes

References genomes enable to associate markers with the trait (e.g., GWAS), to a specific region on the genome. This in turn can be used to find candidate genes in the associated locus (Aranzana et al. 2019). Within the QTL for LCC there are 400 genes. Among them is an interesting gene, the *chloroplast photosystem II stability/assembly factor* (gene id: Prudu003041). Additionally, the QTL for fruit size encompasses only 55 candidate genes (Fig. 4). Two interesting genes found among these 55 genes include the *Auxin Response Factor 2* (*ARF2*) (gene id: Prudu_004998), known as a transcription factor which regulates the auxin effect on diverse developmental process in plants (Liu et al. 2018). The second interesting gene is the *PIN domain-like family* (gene id: Prudu005003) which is known as a transporter of auxin. The function of both genes was established as important in the effect of auxin on cell elongation and fruit size (Devoghalaere et al. 2012). Nonetheless, gene annotation is not always precise, and the name of the gene is far from being enough for determining function. Moreover, the step forward from marker in association to physical boundaries in the reference genome should be done with caution. In many cases, the reference genome has major genomics differences between the parents of the studied population. This is especially true when wild species are used as in this case (Oren et al. 2022). Therefore, some validation, like generating a genetic map with the same marker panel and comparison of the resulting order of markers with the order of markers in the reference genome is a must (Cano-Gamez and Trynka 2020; Brukental et al. 2021). Moreover, de novo assembly of the parent genome for generating reliable reference genome for the specific population is also very important (Oren et al. 2022). Likewise, expression experiments (e.g., transcriptome) could cross validate with the genes in the QTL boundaries. Finally, advanced populations (e.g., backcross or F2) will increase recombination frequency and allow to fine map the trait to narrow down and validate the gene of interest.

## Conclusions

This study aims to examine in practice, and not only as a concept, the potential of using wild almond genetic resources as starting material for breeding and genetic research. Such data is important as a tool for the breeder to understand the limitations of using *P. arabica* as an exotic donor. For example, the dominant effect of *P. arabica* kernel bitterness or the lack of functionality of the *P. arabica Sf* allele. Also, how traits will inherit or interact in cross-hybrid populations, as we demonstrated the positive correlation between canopy area to rootstock perimeter and yield. It is also important for genetic research; since the high phenotypic variance enables genetic mapping of essential characteristics. The genetic mapping of traits like LCC and fruit size hopefully will be developed into active genetic markers in future breeding programs, and for future ‘gene hunting’ research to understand the genetic mechanism behind those traits. Finally, utilizing a distinctive genetic background allows us to ask important domestication and evolutional questions, as shown in the SK and Sf results. Considering all this, we think that despite the long generation time and the introduction of bad alleles from the point of view of commercial cultivars, using wild almonds in genetic crosses is essential, and in the long term, it is cost-effective.

## Supplementary information

Below is the link to the electronic supplementary material.

Supplementary file1 (XLSX 16 KB)

Supplementary file2 (XLSX 23 KB)

Supplementary file3 (XLSX 12 KB)

Supplementary file4 (XLSX 13 KB)

Supplementary file5 (PDF 317 KB)

Supplementary file6 (PDF 155 KB)

## Declarations

### Data archiving statement

All data analyzed during this study are published in this article and in the supplemental information, as well as in our previous study (Brukental et al. 2021). Further data will be given upon request from the corresponding author.
