# Supplementary material for: Exploring the wild almond, Prunus arabica (Olivier), as a genetic source for almond breeding
Source: Tree Genet Genomes. 2024 Sep 24;20(5):37. doi: 10.1007/s11295-024-01668-4 (PMC11469977; doi:10.1007/s11295-024-01668-4)
Supplement: Supplementary file 5 — Supplementary file5 (PDF 317 KB) [file 11295_2024_1668_MOESM5_ESM.pdf]

Tree Genetics and Genomes

Exploring the wild almond, *Prunus arabica* (Olivier) as a genetic source for almond breeding

Hillel Brukental[1,2*], Adi Doron-Faigenboim[3], Irit Bar-Ya'akov[1], Rotem Harel-Beja[1], Taly Training[1], Kamel Hatib[1], Shlomi Aharon[1,2], Tamar Azoulay-Shemer[1] Doron Holland[1]

[1]Unit of Fruit Tree Sciences, Institute of Plant Sciences, Agricultural Research Organization, Newe Ya'ar Research Center, Ramat Yishay, Israel

[2] The Robert H. Smith Institute of Plant Sciences and Genetics in Agriculture, Faculty of Agriculture, Hebrew University of Jerusalem, Rehovot, Israel

[3]Department of Vegetable and Field Crops, Institute of Plant Sciences, Agricultural Research Organization, Volcani Center, Rishon LeZion, Israel

Correspondence:
Hillel Brukental

hillel.brukental@mail.huji.ac.il

Online resource 1. Sf genotyping and phenotyping.

a

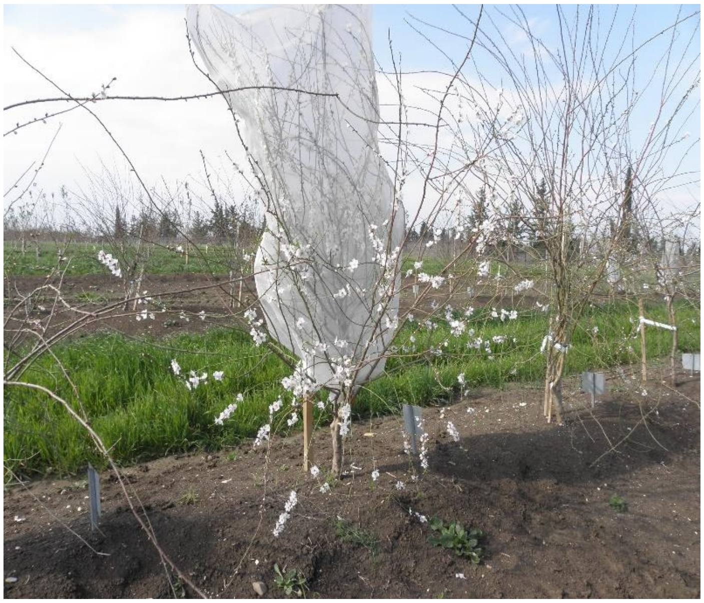

b

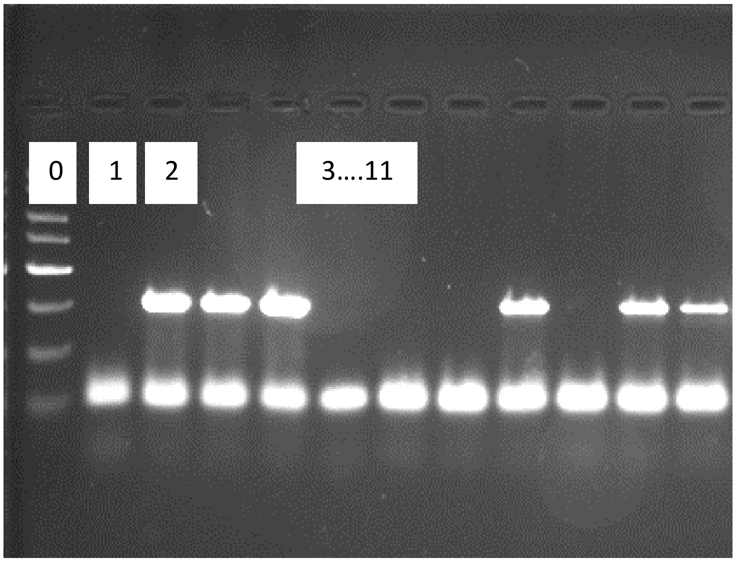

Online resource 1. Sf genotyping and phenotyping. Representative pictures of the self-compatibility screening in the F1 (a). For each individual, a few branches were covered with an insect-proof net during the flowering period to test the self-fertility functionality. Representing agarose gel of the Sf allele genotyping in the F1 population (b). '0' refers to the 'perfect plus' 2kb ladder, '1' represents the negative control (without D.N.A), and '2' illustrates the positive control, which is *P. arabica*. Numbers '3….11' represent individuals from the F1 population. The bend (325 bp) means the presence of the Sf allele (3,4,8,10,11 are positives, and 5,6,7,9 are negatives). The lower bends are some primer dimers. PCR products were separated in a 1% agarose gel.
