# Supplementary material for: Exploring the wild almond, Prunus arabica (Olivier), as a genetic source for almond breeding
Source: Tree Genet Genomes. 2024 Sep 24;20(5):37. doi: 10.1007/s11295-024-01668-4 (PMC11469977; doi:10.1007/s11295-024-01668-4)
Supplement: Supplementary file 6 — Supplementary file6 (PDF 155 KB) [file 11295_2024_1668_MOESM6_ESM.pdf]

Tree Genetics and Genomes

Exploring the wild almond, *Prunus arabica* (Olivier) as a genetic source for almond breeding

Hillel Brukental[1,2*], Adi Doron-Faigenboim[3], Irit Bar-Ya'akov[1], Rotem Harel-Beja[1], Taly Training[1], Kamel Hatib[1], Shlomi Aharon[1,2], Tamar Azoulay-Shemer[1] Doron Holland[1]

[1]Unit of Fruit Tree Sciences, Institute of Plant Sciences, Agricultural Research Organization, Newe Ya'ar Research Center, Ramat Yishay, Israel

[2] The Robert H. Smith Institute of Plant Sciences and Genetics in Agriculture, Faculty of Agriculture, Hebrew University of Jerusalem, Rehovot, Israel

[3]Department of Vegetable and Field Crops, Institute of Plant Sciences, Agricultural Research Organization, Volcani Center, Rishon LeZion, Israel

Correspondence:
Hillel Brukental

hillel.brukental@mail.huji.ac.il

Online Resource 4. QTL mapping results.

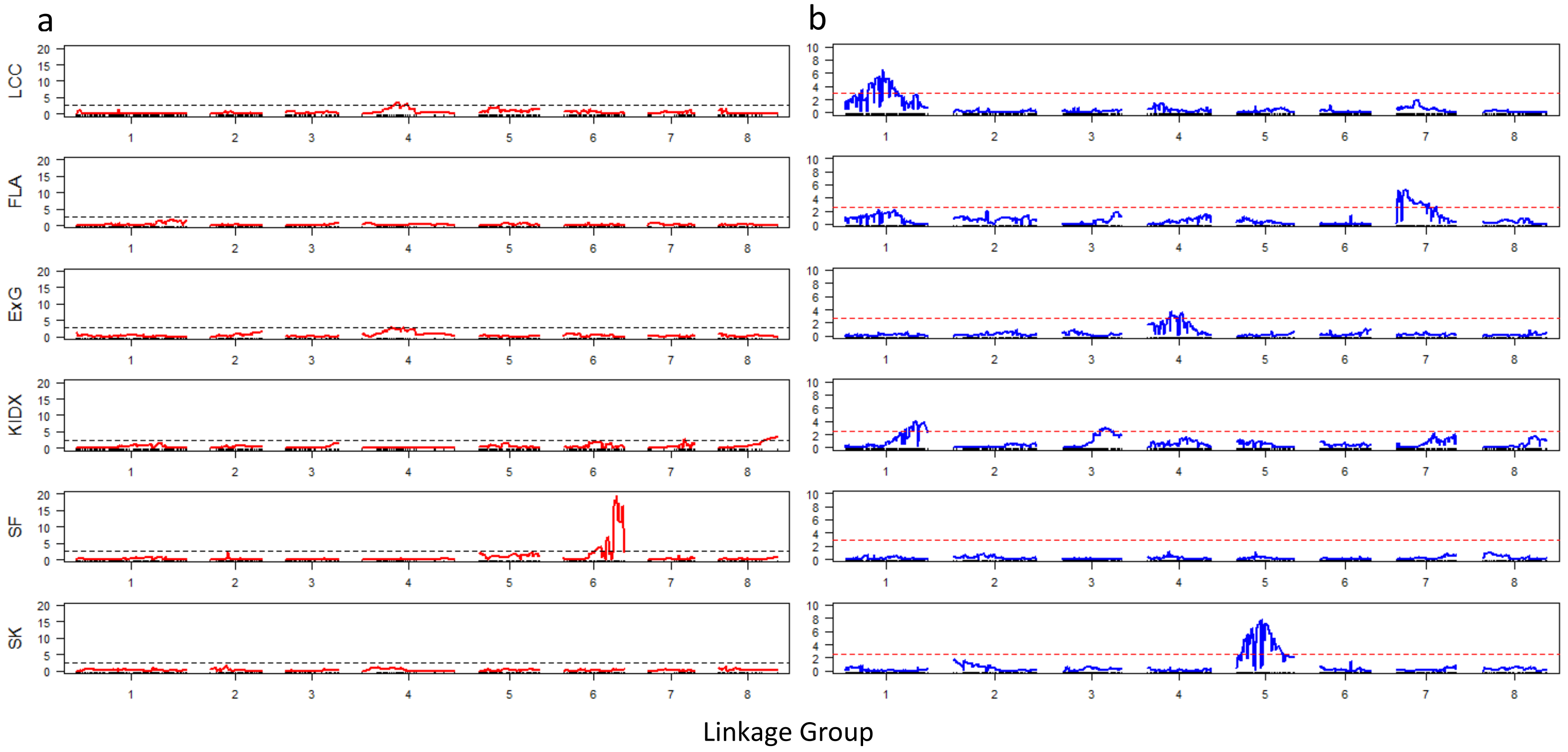

Online Resource 4. QTL mapping of the six segregating traits against the two genetic maps. Red lines represent the QTL mapping of six traits against the *P. arabica* genetic map (a), and blue lines represent the QTL mapping against the UEF genetic map (b). The dashed horizontal line represents the threshold for significance computed by the 1000 permutation test for $\alpha = 0.05$. X-axes are the marker linkage group and its genomic positions (cM). Y axes are the log of odds (LOD) scores.
